# RECQL1 DNA Repair Helicase: A Potential Therapeutic Target and a Proliferative Marker against Ovarian Cancer

**DOI:** 10.1371/journal.pone.0072820

**Published:** 2013-08-09

**Authors:** Sakiko Sanada, Kazunobu Futami, Atsumu Terada, Koji Yonemoto, Sachiko Ogasawara, Jun Akiba, Makiko Yasumoto, Akiko Sumi, Kimio Ushijima, Toshiharu Kamura, Yasuhiro Furuichi, Hirohisa Yano

**Affiliations:** 1 Department of Pathology, Kurume University School of Medicine, Fukuoka, Japan; 2 Gene Care Research Institute Co., Ltd., Kamakura, Japan; 3 Department of Obstetrics and Gynecology, Kurume University School of Medicine, Fukuoka, Japan; 4 Biostatistic Unit, Kurume University School of Medicine, Fukuoka, Japan; Philipps University, Germany

## Abstract

**Objective:**

This study analyzed the clinicopathological correlation between ovarian cancer (OC) and RECQL1 DNA helicase to assess its therapeutic potential.

**Methods:**

Surgically resected OC from 118 retrospective cases, for which paraffin blocks and all clinical data were complete, were used in this study. RECQL1 and Ki-67 immunostaining were performed on sections to correlate RECQL1 staining with subtype and patient survival. Ten OC and two normal cell lines were then examined for RECQL1 expression and were treated with siRNA against RECQL1 to assess its effect on cell proliferation.

**Results:**

Of the 118 cases of adenocarcinoma (50, serous; 26, endometrioid; 21, clear cell; 15, mucinous; 6, other histology), 104 (90%) showed varying levels of RECQL1 expression in the nuclei of OC cells. The Cox hazards model confirmed that diffuse and strong staining of RECQL1 was correlated with histological type. However, RECQL1 expression did not correlate with overall patient survival or FIGO stage. *In vitro*, RECQL1 expression was exceptionally high in rapidly growing OC cell lines, as compared with normal cells. Using a time-course analysis of RECQL1-siRNA transfection, we observed a significant inhibition in cell proliferation.

**Conclusions:**

RECQL1 DNA helicase is a marker of highly proliferative cells. RECQL1-siRNA may offer a new therapeutic strategy against various subtypes of OC, including platinum-resistant cancers, or in recurrent cancers that gain platinum resistance.

## Introduction

Ovarian cancer (OC) represents about 30% of all cancers of the female reproductive system. The mean 5-year survival rate in advanced stage is as low as 31% (FIGO stage IIIC) to 51% (FIGO stage IIIA) (International Federation of Gynecology and Obstetrics (FIGO)). Approximately 75% of patients with advanced (stage III) OC represent with recurrence after surgery, which is fatal [1]. This is largely ascribed to a lack of early warning symptoms and a lack of diagnostic tests that allow early detection of OC and its recurrence [2]. The initial treatment for OC is bulk resection followed by postoperative combination chemotherapy for patients with a stage IC or higher grade of tumor. Thus, the first line treatment is targeted to curative treatment. By comparison, only palliative care is provided for cancer recurrence or in patients who develop chemotherapy resistance. In advanced OC with peritoneal dissemination, while complete regression is achieved, some residual OC cells may promote the emergence of drug-resistant cells under prolonged chemotherapy. Two subgroups of notorious OCs, histologically subclassified as clear cell adenocarcinoma and mucinous adenocarcinoma, are known to be resistant to various anticancer drugs, including the DNA-interacting platinum derivatives. These profiles of OCs represent serious problems among gynecological oncologists.

RECQL1 belongs to a family of DNA helicases, which are ubiquitous enzymes that unwind double-stranded DNA to reveal single-stranded DNA during essential processes, such as replication, transcription or repair. Biochemical and cell biological data show RECQL1 helicase participates in the maintenance of genomic integrity and is highly up-regulated in rapidly proliferating cells, including those in various cancers, such as lung, liver, pancreas, colon, brain and head-and-neck cancers [3–9]. Previously, in the xenograft mouse model of liver cancer, we showed an RNAi therapy that specifically silences the expression of RECQL1 DNA helicase was an effective anticancer treatment and has the potential to be developed as new therapeutic method [10]. Although the efficacy of RECQL1-siRNA has been proven correctly with the cultured cell lines obtained from various cancer origins and also in the xenograft mouse models, OC cells have not so far been studied explicitly [11]. In this paper, we thus analyzed the clinicopathological correlation between ovarian cancer and RECQL1 expression *in vivo* and *in vitro* and make further reference to the therapeutic potential of RECQL1.

## Methods

### Study population and samples

A retrospective search for patients diagnosed with ovarian cancer between 1998 and 2005 was performed using files from the Pathology Department of the Kurume University Hospital, Japan. Written consent was given by the patients for their information to be stored in the hospital database and used for research. The study was approved by the ethical committee of Kurume University School of Medicine. One hundred and eighteen ovarian cancers with matching paraffin blocks were identified. Clinical and pathologic information were reviewed, and the clinical data included age, therapy, FIGO stage and follow-up. Hematoxylin-eosin (H&E) stained slides were reviewed and the diagnosis confirmed independently by two pathologists (SS, HY).

### Immunohistochemical analysis

Four-µm thick serial sections were obtained from selected paraffin-embedded blocks and mounted onto coated glass slides using the BenchMark XT (Ventana Automated Systems, Inc., Tucson, AZ, USA) and ChemMate ENVISION methods (DakoCytomation, Glostrup, Denmark). Immunohistochemistry was performed using mouse monoclonal antibody RECQL1 (Cell Signaling Inc. USA) at a concentration of 6.8 µg/ml, and Ki-67 antibodies that were obtained pre-diluted. Each slide was heat-treated using Ventana’s CC1 retrieval solution for 30 min, and incubated with primary antibodies for 30 min. This automated system used the streptavidin biotin complex method with 3,3’diaminobenzidine as the chromogen (Ventana iVIEW DAB detection kit). We used RECQL1-negative normal and atrophic ovarian stroma of postmenopausal woman (negative control). Some of the OC specimen contained intact ovarian stroma, so that we also could see them as internal negative controls. Scoring was based on the amount and intensity of nuclear RECQL1 staining. The amount was scored as negative (<1% of the positive cells), sporadic (isolated positive cells, but <5%), focal (small cell clusters, but <25% of the positive cells), diffuse (>25% of stained cells). Intensity was graded as light, moderate, or strong intensity. Diffuse and strong staining was regarded as ++, sporadic and focal staining, with any intensity was regarded as +, negative as -. Statistical analysis were carried out for each clinicopathological characteristic among the (-), (+) and (++) groups. Proliferative index was evaluated using Ki-67 positive cell counts per 10 high power fields (Ki-67 labeling index (LI)). Stained tissue sections were scored and examined without knowledge of the clinicopathological data or patient outcome.

### Statistical analysis

The categorical data and RECQL1 expression was examined by the Χ^2^ test. Ki-67 LI and RECQL1 expressions were examined by Wilcoxon rank-sum test. Histological grade and RECQL1 expression were evaluated by exact Cochran-Armitage trend test. Cox proportional model in a conditional logistic regression was performed using FIGO stage and RECQL1 expression. Log-rank test with Kaplan–Meier model was used to compare survival populations between RECQL1 expressions. All tests were 2-sided, and a *P* value of <0.05 was considered statistically significant. Statistical analyses were conducted using Statistical Analysis Software (SAS) version 9.2 (SAS Institute, Cary, NC).

### Cells and culture conditions

In this study, we used 10 human OC cell lines (Table 1) OVCAR-3, OVCAR-5, SK-OV-3, TOV-21G, TOV-112D and ARPE-19 were obtained from ATCC (Manassas, VA, USA). TIG-3 and MCAS were obtained from Health Science Resource Bank (Osaka, Japan). KOC-2S, KOC-3S, KOC-5C and -7C were established in Kurume University School of Medicine [12]. The cells were grown in Dulbecco’s modified Eagle’s or RPMI-1640 medium (Nacalai Tesque, Kyoto, Japan) and were incubated at 37° C in a humidified chamber supplemented with 5% CO_2_.

**Table 1:** Types of Cell Lines and Sites of Establishment.

**Cell line**	**Cell type**	**Site of establishment**
OVCAR-3	Serous adenocarcinoma	ATCC *
OVCAR-5	Serous adenocarcinoma	ATCC
KOC-2S	Serous adenocarcinoma	Kurume university
KOC-3S	Serous adenocarcinoma	Kurume university
KOC-5C	Clear cell adenocarcinoma	Kurume university
KOC-7C	Clear cell adenocarcinoma	Kurume university
SK-OV-3	Clear cell adenocarcinoma	ATCC
TOV-21G	Clear cell adenocarcinoma	ATCC
TOV-112D	Endometrioid adenocarcinoma	ATCC
MCAS	Mucinous adenocarcinoma	Health Science Resource Bank **
APRE-19	Normal cell for control †	ATCC
TIG-3	Normal cell for control ‡	Health Science Resource Bank

† normal spontaneously arising retinal pigmentation epithelium cell line.

‡ normal lung diploid fibroblasts from human fetuses.

* ATCC, Manassas, VA, USA.

** Health Science Resource Bank: Osaka, Japan.

### Immunoblotting

The protein levels of RECQL1 and other proteins participating in the cellular DNA damage repair system were monitored by using immunoblotting. The cells were washed with ice-cold phosphate buffered saline (PBS), pelleted by centrifugation and then lysed in a sodium dodecyl sulfate (SDS) buffer containing 1% SDS, 2% β-mercaptoethanol, 20% glycerol, 30 mM Tris-HCl (pH 6.8) and 0.2 M dithiothreitol. The cell lysate was boiled for 10 min and then was electrophoresed on 5-20% gradient SDS-polyacrylamide gels. Proteins fractionated on the gels were electrophoretically transferred to polyvinylidene difluoride membranes (Immobilon, Millipore, MA, USA) and were blocked overnight with 5% skimmed milk in PBS. The membranes were then incubated with either anti-RECQL1 monoclonal antibody Q1N3 (Cell Signaling Technology, Tokyo, Japan) or anti-β-actin monoclonal antibody (ICN Biomedicals, Aurora, OH, USA) for 60 min at room temperature. The membranes were washed with 0.05% Tween-20 in PBS, incubated with anti-mouse IgG conjugated with horseradish peroxidase (DakoCytomation, Carpinteria, CA, USA) and washed. Membranes were subsequently developed using an enhanced chemiluminescence reagent (ECL Plus, Amersham Biosciences, UK).

### siRNA and RNA interference

siRNAs (21 bp) targeting RECQL1 mRNA (RECQL1-siRNA) and negative control siRNA (NS-siRNA) were chemically synthesized (GeneDesign, Inc, Osaka, Japan). All siRNA sequences had an overhanging 3'-dTdT at the 3' end [11]. Sequence-specific gene silencing was confirmed using cDNA microarray analysis with the Affymetrix GeneChip system (Human Genome U133 Plus 2.0 Array). For transfection at 24 h after plating, the cells were incubated with 30 nM siRNA duplex for 4h in the presence of RNAiMAX (Invitrogen, Carlsbad, CA, USA), diluted 2-fold with the serum containing fresh medium, and were cultured at 37 °C for indicated terms. Total RNA was extracted from cultured cells at various time points using an RNeasy Mini Kit (Qiagen GmbH, Hilden, Germany) according to the manufacturer’s protocol after chemical compounds treatment. Reverse transcriptase-PCR analyses were carried out using the ABI PRISM 7000 Sequence Detection System with TaqMan probes and primers (ABI, Foster, CA, USA). The β-actin gene was used as an internal standard (TaqMan probe ID; 431088E; ABI). Cell proliferation was measured at 24, 48, 72, 96 and 120 hours by fluorescent assay using Cell Titer-Glo Luminescent (Promega, WI, USA) according to a protocol. As for cell cycle analysis, the cells were transfected with RECQL1-siRNA or with NS-siRNA under the same conditions described above, and were incubated for 72 hours at 37 °C. Cells and cell debris were collected, washed with PBS and were fixed in ice-cold methanol for 2 hours. Then, the cells were treated with pancreatic RNase A (Nippon Gene, Toyama, Japan), stained with propidium iodide (Sigma, St Louis, MO, USA) for 30 minites, and analyzed by flow cytometry. Fluorescence was measured using EPICS XL (Beckman Colter, Tokyo, Japan). For each sample, 7000 events were analyzed. These analyses were repeated 3 times for each cell lines. The stage of cell cycle and the population of cell are shown with the mean ± SD values in each flow cytometric profile.

## Results

### Clinical and microscopic findings

The age range for ovarian cancer patients was from 20 to 82 (mean, 57.1) years. All 118 patients underwent total hysterectomy, bilateral salpingo-oophorectomy, omentectomy and lymph node dissection or sampling. Surgically resected specimens revealed that 50 patients were serous adenocarcinoma, 26 were endometrioid adenocarcinoma, 21 were clear cell adenocarcinoma, 15 were mucinous adenocarcinoma (expansile invasion and invasive mucinous adenocarcinoma), and 6 were of other histology, including 3 squamous cell carcinoma, 2 mixed carcinoma (squamous cell carcinoma and serous adenocarcinoma, or squamous cell carcinoma and clear cell adenocarcinoma) and 1 transitional cell carcinoma.

### Immunohistochemical results

**Figure 1:**
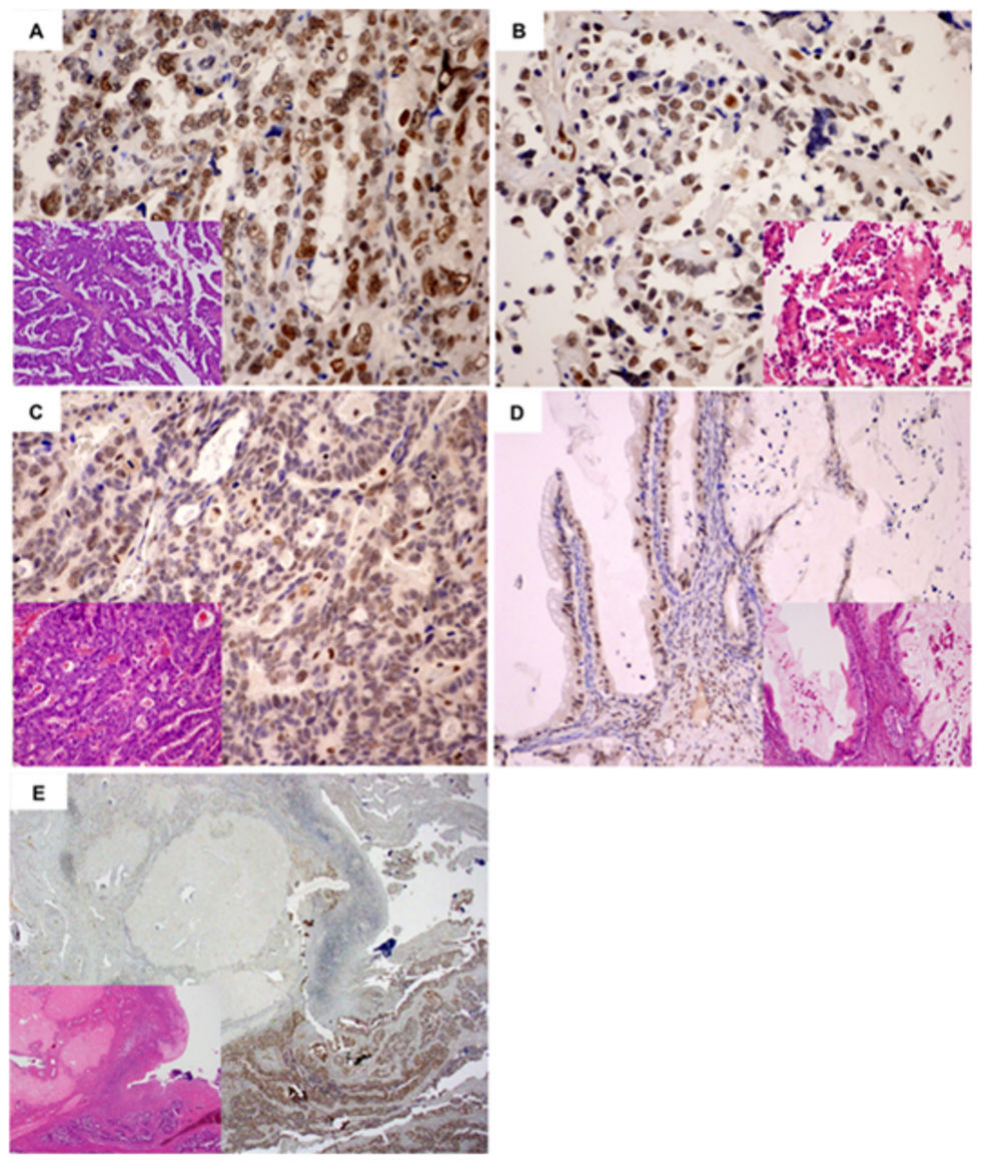
Histological features and RECQL1 **(++) expression in ovarian cancers.** (A) Serous adenocarcinoma, (B) Clear cell adenocarcinoma (C) Endometrioid adenocarcinoma, (D) Mucinous adenocarcinoma and (E) intact ovarian stroma as negative control (inset: hematoxylin and eosin staining).

**Figure 2:**
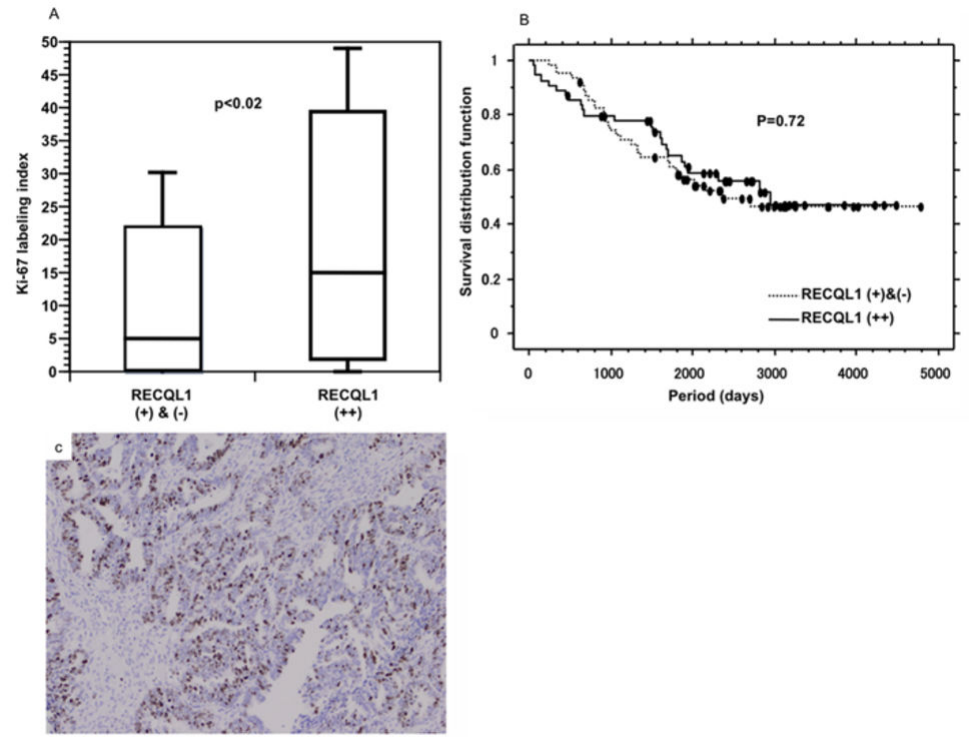
Tumor cell proliferation index and survival estimate. (A) Correlation between Ki-67 labeling index and RECQL1 (++) cases. Photomicrograph illustrates high Ki-67 staining in the serous adenocarcinoma. RECQL1 (++) cases were significantly associated with high Ki-67LI (p=0.02). (B) Kaplan-Meier survival estimate between RECQL1 (++) and (+), (-) cases. By log-rank analysis, there was no significance between RECQL1 expression and overall survival (p=0.72).

Immunohistochemical results were available for 118 surgically resected OCs. RECQL1 expression was observed in the nuclei of OC cells to various extents in 104 (90%) of the 118 OC specimens (serous type, 94%; endometrioid type, 89%; clear cell type, 81%; mucinous type, 87%; and others, 100%). Based on amount and intensity, 55 cases were regarded as (++), for which 30 cases (54.5%) were of serous type, 9 (16.3%) of endometrioid type, 9 (16.3%) of clear cell type, 3 (5.4%) of mucinous and 4 (7.2%) of others (Figure 1). Sixty-cases were regarded as (+) and (-), for which 20 (31.7%) were of serous type, 17 (27.0%) of endometrioid type, 12 (19.0%) of clear cell type, 12 (17.5%) of mucinous and 2 (1.6%) of others. RECQL1-(++) expression in surgically resected tissues has a propensity for being observed in serous type, and less frequently in endometrioid, clear and mucinous types (p<0.03) (Table 2). Ki-67 labeling index (LI) revealed the highest proliferative potential in serous adenocarcinoma, with an average of 20.8% (endometrioid, 15.8%; clear cell, 7.3%; and mucinous, 9.4%). RECQL1-(++) cases were significantly associated with high Ki-67LI (p=0.02) (Figure 2A).

**Table 2:** Scored Immunohistochemical RECQL1 Expression and Relationship to Histologic Type and FIGO Stage.

**Scored RECQL1Expression**	(-) and (+)	(++)	
**Parameters**			
**Histological type^a^**			
serous type	20 (31.7%)	30 (54.5%)	
endometrioid type	17 (27.0%)	9 (16.3%)	
clear cell type	12 (19.0%)	9 (16.3%)	
mucinous type	12 (17.5%)	3 (5.4%)	
others	2 (1.6%)	4 (7.2%)	
**FIGO stage^b^**			
lower stage (Stage I and II)	60 (50.8%)	45 (38.1%)	
advanced stage (Stage III and IV)	3 (2.5%)	10 (8.5%)	

^a^ RECQL1 expression was significantly (p<0.03) correlated with histological type.

^b^ p<0.02 vs. advanced stage, although it lost predictive value when adjusted by histological type.

### Inclusion of clinical factors

A Cox hazards model confirmed that diffuse and strong staining of RECQL1 (HR 2.3) was correlated with histological type, high Ki-67 LI and advanced FIGO stage, although FIGO stage lost its predictive value when adjusted by histological type (Table 2). By log-rank analysis, there was no significant association between RECQL1 expression and overall survival (p=0.72) (Figure 2B).

### RECQL1 expression levels in OC cell lines

**Figure 3:**
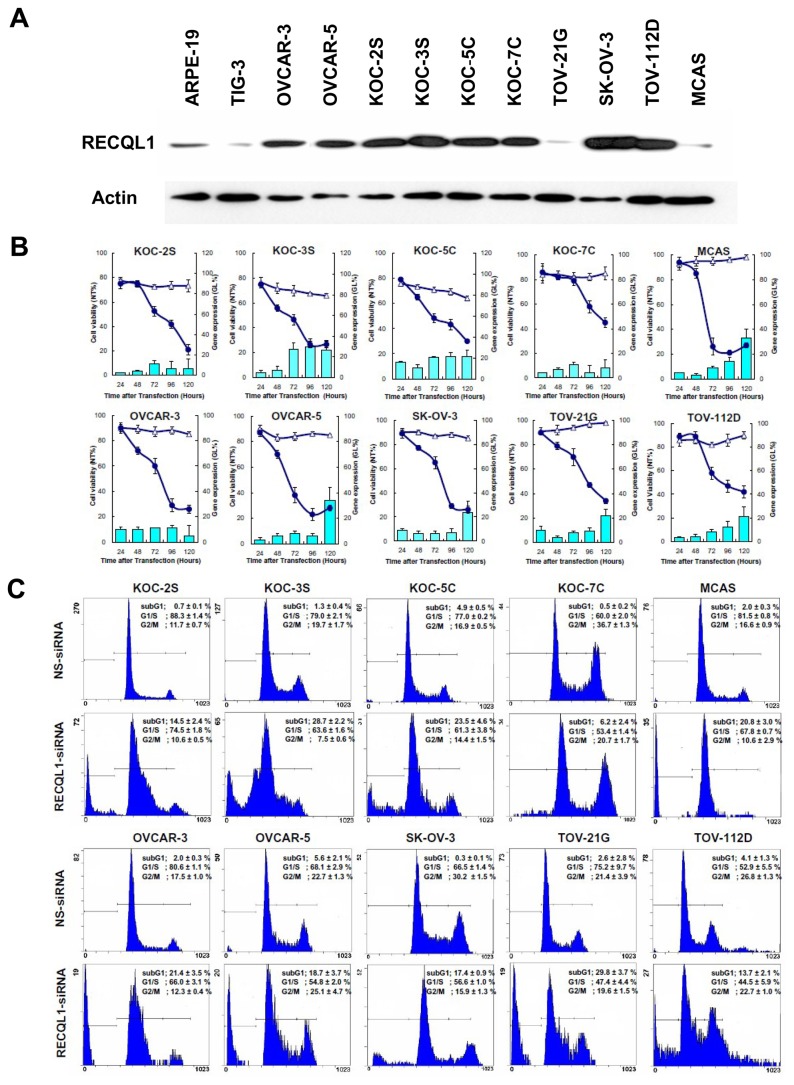
In vitro analysis on RECQL1 expression. (A) RECQL1 expression levels in ten OC cell lines. RECQL1 expression was found to be exceptionally high in KOC-2S, KOC-3S, OVCAR-3, OVCAR-5, KOC-7C, SK-OV-3 and Tov-112D ovarian cancer cell lines as compared with the two normal cell lines (ARPE-19 and TIG-3). (B) The effect of RECQL1 silencing on the growth of OC cell line by time-course analysis. A total of ten OC cell lines were transfected with either RECQL1-siRNA (solid circle) or NS-siRNA (triangle) for a period of 120 hours as described in the Materials and Methods. Cell viability decreased with time after siRNA treatment. RECQL1-siRNA significantly inhibited the proliferation of a wide range of OC cell lines in accordance with the reduction of RECQL1 mRNA (shown as histograms). During the time-course, RECQL1 mRNA expression was reduced differentially, depending on cell line. Data represent the means ± SD, n = 3. (C) The effect of RECQL1 silencing on the cell cycle of OC cell line measured by flow cytometric analysis. Cell lines were transfected with either NS-siRNA (Upper panels) or RECQL1-siRNA (lower panels) for 72 hours. The percentage of cell populations in the subG1, G1/S and G2/M stages are shown as mean ± SD in each flow cytometric profile.

As a greater amount of RECQL1 immunoreactivity was observed in surgically resected OC specimens, we investigated RECQL1 expression using various OC cell lines. As controls, normal cells ARPE-19 and TIG-3 were similarly characterized. Most, if not all, cancer cells examined so far showed higher expression level of RECQL1 helicase protein as compared with the normal cells (Figure 3A). RECQL1 expression was found to be exceptionally high in the rapidly growing cell lines, such as KOC-2S, KOC-3S, OVCAR-3, OVCAR-5, KOC-7C, SK-OV-3 and TOV-112D cell lines. Interestingly, TOV-21G and MCAS showed a similar low expression to the normal TIG-3 cell line.

### Inhibition of OC cell proliferation by RECQL1 silencing

By immunoblotting, all ten OC cell lines were found to express greater amount of RECQL1 helicase protein than normal cells. Based on these results, we investigated the effect of RECQL1 silencing on the growth of various OC cell lines by a time-course analysis. The 10 OC cells lines and the 2 normal cells lines were transfected either with RECQL1-siRNA or with NS-siRNA (triangle) for 120 hours. As shown in Figure 3B, RECQL1-siRNA (solid circle) significantly inhibited the proliferation of a wide range of OC cell lines in accordance with the reduction of RECQL1 mRNA (shown as histograms), while it had no effect on the growth of the two normal cell lines (data not shown in this paper) [10]. Cell viability, shown as the relative growth as compared with that of non-treated cells, decreased with time following siRNA treatment. During the 24-120 hour time-course, RECQL1 mRNA expressions were reduced differentially depending on the cell line. Interestingly, the level of RECQL1 mRNA increased toward the end of the time-course for a few of the cell lines, although this increase never went beyond 40% of the original expression.

In regard to the mechanism underlying the reduced cell viability, we investigated the cell cycling feature of several RECQL1-siRNA treated OC cells. The siRNA-treated cells (post-transfection 72 hrs) were isolated from culture and were subjected to the cell cycle analysis. As shown in Figure 3C, the cell populations of all the RECQL1-siRNA-treated OC cells contained a distinct subG1 cell fraction of dead cells, and an accumulation of cells in the diffused G1/S and G2/M phases, showing a characteristic mitotic cell death occurring during cell cycle (lower panels). In some RECQL1-siRNA-treated OC cells, the increased populations of G1 and G2/M phases, cells indicative of a cell cycle arrest due to DNA damage checkpoint system. By contrast, no such abnormal features are seen with the cognate OC cells treated with the non-silencing siRNA used for the control experiments (upper panels). Thus, the apparent growth inhibition of cancer cells (seen in Figure 3B) is not due to the retardation of cell growth but is due to killing of viable OC cells and reduction of cell number caused by mitotic cell death.

## Discussion

For both serous and endometrioid adenocarcinoma, combination therapy with carboplatin and paclitaxel is an effective strategy and gives rise to an excellent therapeutic result, even if the cancer is found at an advanced stage. In contrast, there is no curative regimen against platinum-resistant OCs, such as clear cell adenocarcinoma and mucinous adenocarcinoma, or recurrent OCs of a refractory type. Clear cell adenocarcinoma and mucinous adenocarcinoma show indolent behavior when they are confined to the ovary and are often diagnosed at an early stage, which confers a better prognosis for the patient. However, once the disease extends into the extra-ovarian space, their cure rates are much lower than that of serous adenocarcinoma and endometrioid adenocarcinoma [13,14]. Platinum agents as a standard therapeutic regimen include carboplatin, which targets DNA synthesis at S-phase and induces apoptosis. Paclitaxel, by comparison, interferes with the synthesis of tubulin to contribute to cell cycle arrest. For this reason, these chemotherapeutic agents function most effectively in active, proliferative tumors, whereas slow-growing, low-Ki-67 LI tumors, such as clear cell adenocarcinoma and mucinous adenocarcinoma, do not respond well, and treatment with these therapies may result in hyposensitivity.

To date, several trials to identify alternative chemotherapeutic or molecular-targeted agents have been made against platinum-resistant OCs; however, a general consensus on the most optimal first-line therapy has not been reached [15–20]. Moreover, studies show that a considerable number of patients with recurrence for carcinomas that were originally platinum-sensitive, such as serous adenocarcinoma and endometrioid adenocarcinoma, end up with platinum-resistant carcinomas.

It has been reported that RECQL1 expression is observed in liver, colorectal, lung cancer, glioblastoma and head-and-neck cancers [7,21]. In this study, using surgically resected OC specimens, we observed nuclear RECQL1 expression in 90% of the 4 major subtypes of OC (serous, endometrioid, clear cell and mucinous type), with high (++) expression seen in 54.5% of cases. A comparison between RECQL1 expression and Ki-67 LI indicated that RECQL1 (++) group was associated with high Ki-67LI (p=0.02); thus, RECQL1 may be a useful proliferative molecular marker. We previously reported that RECQL1 positivity in hepatocellular carcinoma was correlated with histological grade and Ki-67 LI [10]. Thus, RECQL1 expression may suggest be strongly correlated with tumor progression and differentiation. In terms of tumor progression, RECQL1 expression was significantly associated with FIGO stage distribution (p=0.01), although it lost its value when adjusted for histological type. Therefore, while RECQL1 expression may act as a proliferative parameter, we failed to prove associations between RECQL1 and FIGO stage and overall survival. We speculate that this may be why the size of the residual disease correlates most clearly with survival in patients with an advanced stage tumor [22].

In our *in vitro* analysis, we investigated the potential anticancer effect of RECQL1-silencing in cell lines that are representative of serous, endometrioid, clear cell, and mucinous adenocarcinomas. Most, if not all, cancer cells examined so far showed higher expression levels of RECQL1 helicase protein as compared with the normal cells. Considering that RECQL1 is highly up-regulated in rapidly growing cells, including various cancers and transformed cells [4–6,8], clear cell and mucinous adenocarcinoma which are known to relatively slow growing OC, showed less RECQL1 expression may be reasonable. In such slow-growing cancer cells, the amount of DNA repair enzymes including RECQL1 helicase may be spared, perhaps because the ample time provided by slow growth instead helps to fulfill any necessary tasks required in DNA repair.

Previously we showed antiproliferative effect by RECQL1-siRNA in various cancer cell lines [10,21]. This is resulted from an increased accumulation of DNA damage in cancer cells caused by RECQL1 silencing led to a M phase arrest and cell death because of incomplete checkpoint systems, which indicative of mitotic catastrophe [10,21]. On the other hand, normal cell lines, including TIG-3 and APRE-19, showed tolerance to mitotic catastrophe by RECQL1 silencing [10,21]. Main difference between normal cells and cancer cells would be explained by ability in cell cycle control. Most cancer cells are deficient in G1 and G2 checkpoint function and thus fail to arrest the cell cycle at G1 and G2 phases to permit cell to engage in DNA repair. Instead, the cells proceed in the cell cycle to M phase where DNA repair is not permitted. Thus, cells eventually undergo cell death as they enter mitosis [23–25]. In contrast, the checkpoint systems in normal cells remains intact, which resulting in transient cell-cycle arrest until the DNA problem is resolved by recruiting an appropriate repair system [21]. In other words, it can be regarded as normal cells are resistant to RECQL1 silencing, while the checkpoint defective cancer cells are not.

In this study, we also showed RECQL1-siRNA silencing induced cell death and inhibited cell proliferation in OC cell lines. Our previous data would support that decreased OC cell viability was also resulted from mitotic catastrophe by RECQL1-siRNA silencing. More or less, RECQL1 expression was observed for all histological subtypes of OC, which may suggest an efficacy of RECQL1-siRNA on platinum-resistant cancers, such as clear cell adenocarcinoma, mucinous adenocarcinoma and some of recurrent cancers with platinum-resistant type.

A synergistic anti-cancer effect was observed when RECQL1-siRNA was locally administered into mice with head-and-neck tumors together with an intravenous injection of a cis-platinum derivative [14]. The results suggested the potential participation of RECQL1 DNA helicase in the reduction of cis-platinum-induced DNA damage. Regarding the therapeutic application of siRNA against malignant tumors, several investigators have reported the efficacy of a combination of other chemotherapeutic agents such as gemcitabine [26], camptothecin [27] and adriamycin [28]. The results of these studies indicated that such a combinatory therapy could reduce the adverse effects of the chemotherapeutic agents while increasing the sensitivity of the cancer cells. Comparatively, many other chemotherapeutic agents tend to damage non-cancerous tissue. For example, bone marrow suppression, peripheral nerve disorder, cardiotoxicity and nephrotoxicity are often induced.

Recently, heterogeneous gene expression in cancer tissues was reported via studies on the consecutive renal tumor-biopsy specimens [29]. Even in the same tumor sample, different gene expression signatures were detected by different sample areas and primary versus metastatic sites [29]. So far, numerous molecular targets have been identified for anticancer drug development. From the point of view of mutations or differential expressions of genes during cancer development, it is imperative that the most indispensable gene required for cancer growth is targeted. Compared with other molecular targeted therapies, RECQL1-siRNA only targets DNA repair, and acts regardless of any gene mutation or expression signature change specific to the cancer. We believe that RECQL1 is one of just a few candidate gene targets that do not affect the normal cell populations.

In conclusion, RECQL1 should be considered as a new proliferative marker. Furthermore, RECQL1-siRNA may have the potential to be applied as a new therapeutic method against OC of various histological and clinical characteristics, including those of a platinum-resistant subtype.
